# Association between physical fitness and musculoskeletal health in firefighters

**DOI:** 10.3389/fphys.2023.1210107

**Published:** 2023-07-04

**Authors:** Jaron Ras, Elpidoforos S. Soteriades, Denise L. Smith, Andre P. Kengne, Lloyd Leach

**Affiliations:** ^1^ Department of Sport, Recreation and Exercise Science, Faculty of Community and Health Sciences, University of the Western Cape, Cape Town, South Africa; ^2^ Healthcare Management Program, School of Economics and Management, Open University of Cyprus, Nicosia, Cyprus; ^3^ Environmental and Occupational Medicine and Epidemiology (EOME), Department of Environmental Health, Harvard T.H. Chan School of Public Health, Boston, MA, United States; ^4^ Health and Human Physiological Sciences, Skidmore College, Saratoga Springs, NY, United States; ^5^ Non-Communicable Diseases Research Unit, South African Medical Research Council, Cape Town, South Africa

**Keywords:** firefighting, discomfort, injury, workload, physical activity, strength, endurance, cardiorespiratory

## Abstract

**Introduction:** Firefighters are often placed in situations that require high levels of physical exertion, leading to significant strain on firefighters’ musculoskeletal system, predisposing them to musculoskeletal discomfort (MSD) and/or musculoskeletal injury (MSI). Physical fitness programs are often recommended and justified, in part, to prevent injuries. The aim of this study was to determine the association between physical fitness and musculoskeletal health (MSH) in firefighters.

**Methods:** A total of 308 full-time firefighters took part in the study conducted in Cape Town, South Africa. Physical fitness tests encompassed a non-exercise estimation for cardiorespiratory fitness, grip and leg strength for upper and lower body strength, push-ups and sit-ups for muscular endurance, and sit-and-reach for flexibility. The Nordic Musculoskeletal Questionnaire and Cornell Musculoskeletal Discomfort Questionnaire were used to determine MSIs and MSD, respectively. A *p*-value <0.05 indicated statistical significance.

**Results:** Every one-unit increase in AbVO2max, push-ups, sit-ups, and sit-and-reach decreased the odds of firefighters reporting MSIs by 5% (*p* = 0.005), 3% (*p* = 0.017), 3% (*p* = 0.006), and 3% (*p* = 0.034), respectively. Every one repetition increase in push-up capacity increased the odds of firefighters reporting neck, elbow and forearm, wrist and hand, and thigh discomfort by 3% (*p* = 0.039), 4% (*p* = 0.031), 5% (*p* = 0.002), and 5%` (*p* = 0.007), respectively. Every one repetition increase in sit-up capacity increased the odds of firefighters reporting upper back discomfort and thigh discomfort by 5% (*p* = 0.045) and 7% (*p* = 0.013), respectively.

**Conclusion:** Maintenance of physical fitness is likely beneficial in reducing MSIs, which, however, may increase the feeling of MSD in firefighters. In addition, it may be noticed that there is an ideal level of physical fitness that is conducive to the reduction of MSIs and should be studied further.

## 1 Introduction

Firefighting is globally acknowledged to be a dangerous occupation, routinely placing firefighters in hazardous situations that often require high levels of physical exertion, such as fire suppression, victim rescue, and door breaches (Smith et al., 2013; Smith et al., 2019). These workplace stressors place firefighters at high risk for sustaining serious and, sometimes, career-ending injuries (Poston et al., 2011; Orr et al., 2019). In addition, firefighters are regularly exposed to hazardous chemicals and fumes and high temperatures (Frost et al., 2015a; Smith et al., 2019; Nazari et al., 2020a). Due to the hazards of the profession, firefighters are required to wear heavy and insulated personal protective equipment (PPE) that places additional strain on an already burdened musculoskeletal system (Smith et al., 2013; Smith et al., 2016). As a consequence of the physically exhaustive nature of the profession, which often challenges their abilities to perform their work safely, fire departments have recommended firefighters to engage in regular and structured physical activity to manage these various stressors and workplace hazards (Poplin et al., 2013; Poplin et al., 2016; Nowak et al., 2018).

Frost et al. (2015a) reported that injuries occurring at the fire station (37.9%), during physical training (26.6%), during fire emergencies (14.7%), and non-fire emergencies (12.1%) were the most frequent ones. In addition, injuries and injury-related absenteeism are costly to fire departments and municipalities (Poston et al., 2011). Furthermore, a study reported that only 1%–5% of duty time was spent in fire suppression activities (Kales et al., 2007). However, injuries of higher severity occurred more frequently on the fireground (Poplin et al., 2012). Moreover, higher total number of hours worked by firefighters showed a higher incidence rate of injuries (Poplin et al., 2012). To perform their duties aptly, firefighters are required to maintain all aspects of physical fitness (Williford et al., 1999; Rhea et al., 2004; Michaelides et al., 2008; Chizewski et al., 2021), often through occupational specific exercise interventions (Andrews et al., 2019; Chizewski et al., 2021). Previous studies have indicated that a linear relationship existed between physical fitness and work performance (Williford et al., 1999; Rhea et al., 2004; Chizewski et al., 2021). Injury incidence has been related to increasing age and more years of experience as firefighters (Hong et al., 2012; Frost et al., 2016; Yoon et al., 2016). Studies have shown that firefighters tend to become less active as they age and, along with the attrition of the musculoskeletal system, which is associated with both an increase in age and years of experience, as a product of their work, are significantly predisposed to injuries (Hong et al., 2012; Frost et al., 2015a; Yu et al., 2015). Injuries, particularly moderate-to-severe injuries, result in substantial loss of time from work and, due to medical expenses, become costly to fire departments (Poston et al., 2011; Frost et al., 2016). To reduce the incidence of injuries, it is suggested that firefighters remain physically active in their leisure time or when off-duty (Poplin et al., 2013; Frost et al., 2015a; Nowak et al., 2018), and many fire departments schedule prescribed exercise programs when firefighters are on duty (Vaulerin et al., 2016), though studies have shown that higher overload in workload may predispose firefighters to injury (Vaulerin et al., 2016; Ras and Leach, 2022). This suggests that monitoring the overall weekly workload may be beneficial for firefighters, given the physical nature of their occupation (Poplin et al., 2013; Yu et al., 2015).

Physical fitness has been related to lower the incidence of musculoskeletal injuries (MSIs) in firefighters (Poplin et al., 2013; Poplin et al., 2016). Systematic reviews support the aforementioned finding, where it has been reported that cardiorespiratory fitness, muscle strength, muscular endurance, and flexibility were significantly related to reduction in injuries (de la Motte et al., 2017; Lisman et al., 2017; de la Motte et al., 2019). On the other hand, high weekly duty workloads may be related to insufficient time for recovery among firefighters (Vaulerin et al., 2016; Ras et al., 2022a; Ras and Leach, 2022). Monitoring of overall workload may allow fire departments to adjust the level of total physical activity firefighters are engaged in, either occupational activity or recreational activity, adjusting the workload to allow for more time for rest and recovery, thus reducing the likelihood of overload-related musculoskeletal discomfort (MSD) and/or MSIs (Yu et al., 2015; Bustos et al., 2022; Giuliani-Dewig et al., 2022). It is plausible that higher physical fitness would relate to lower feelings of MSD and pain in firefighters (Azmi and Masuri, 2019; Kodom-Wiredu, 2019; Nazari et al., 2020a). However, it is also possible that higher levels of physical fitness may cause firefighters to exert themselves more vigorously during emergency operations, thereby triggering workload-related feeling of MSD and chronic pain, and this may be exacerbated by repetitive movements (Rintala et al., 2015). Higher physical fitness has been shown to be related to improved occupational performance in firefighters, and it is logical to assume that increased levels of physical activity and physical fitness would also provide an additional benefit of better musculoskeletal health (MSH) (Ras et al., 2022a; Ras et al., 2022b).

It has been reported that firefighters in South Africa have high workloads while on duty, while many firefighters are physically inactive during their leisure time (Ras and Leach, 2022). However, there are firefighters in South Africa who are remarkably physically active both on-duty and off-duty (Ras et al., 2022c; Ras and Leach, 2022), which could place this population at an increased risk of reporting MSD or sustaining MSIs while on duty. It is plausible that firefighters who engage in high levels of duty-related physical activity, but are insufficiently active in their leisure time, (Frost et al., 2015a; Poplin et al., 2016) and those who engage in high levels of both duty-related physical activity and leisure time physical activity could be equally predisposed to MSIs (Vaulerin et al., 2016; Ras and Leach, 2022), possibly due to the mismatch between physical fitness and job tasks, and also due to overload of the musculoskeletal system. There has been insufficient research on the association between physical fitness and MSH in firefighters, particularly in South Africa. The South African Fire and Rescue Services policy on physical fitness is devoid of established guidelines requiring firefighters to remain physically active or maintain a fitness standard, perhaps, in part, due to the lack of research on this population. Therefore, the aim of this study was to determine the association between physical fitness and MSH (MSIs and MSD) in firefighters in the City of Cape Town Fire and Rescue Service (CoCTFRS).

## 2 Methods

### 2.1 Study design and population

This cross-sectional study recruited 308 firefighters from the CoCTFRS between June and August 2022. Physical testing was used to acquire information on physical fitness (cardiorespiratory fitness, muscular strength and endurance, flexibility, and body composition), and a researcher-generated questionnaire, which included two validated questionnaires, was used to acquire information on MSH (MSIs and MSD). All volunteers for this study provided written informed consent before inclusion. Due to injury or inability to perform the physical fitness tests, the total number of firefighters who completed the physical fitness assessment was reduced to 304 for the grip strength, leg strength, push-up, sit-up, and sit-and-reach tests. The study was approved by the University of the Western Cape Biomedical Research Ethics Committee (BM21/10/9) and authorized by the Chief Fire Officer and the Department of Policy and Strategy. A detailed description of the methods used is available in Ras et al. (2022c).

### 2.2 Sampling and participant recruitment

Data collection took place at a standardized fire station in the metropolitan area of the City of Cape Town during the CoCTFRS’s yearly physical fitness evaluation. Every third firefighter from the 96 platoons (32 fire stations) was selected to participate. Each of the 96 firefighting platoons was made up of eight–twelve firefighters. All full-time firefighters between the ages of 20 and 65 who were on active duty during the time of testing were considered. Firefighters who were on administrative duty or sick leave, firefighters who were removed from active duty due to injury, and those who worked part-time or seasonally were disqualified from participating in the study.

### 2.3 Physical fitness measures summary

Physical fitness was measured in accordance with the American College of Sports Medicine (ACSM) guidelines (Liguori et al., 2021). Cardiorespiratory capacity was calculated using a validated non-exercise calculation (Rexhepi and Brestovci, 2014; Ras et al., 2022c) to estimate oxygen consumption (VO_2_). The push-up and sit-up tests were used to assess muscular endurance; handgrip and leg strength tests were used to assess upper and lower body muscle strength, respectively; and the sit-and-reach (YMCA sit-and-reach protocol (Liguori et al., 2021)) test was used to assess flexibility. Body mass and lean body mass (LBM) were used as a measure for body composition and assessed using a Tanita© (Tanita©, Tokyo, Japan) BC-1000 Plus bioelectrical impedance (BIA) analyzer. Briefly, for the push-up and sit-up tests, firefighters were required to perform as many push-ups and sit-ups within a minute until volitional fatigue or failure (Liguori et al., 2021). Grip strength was measured using a Takei® 5401-C handgrip dynamometer and leg strength using a Takei® back and leg strength dynamometer, following standardized protocols and with three attempts, with the highest value being recorded (Liguori et al., 2021). The sit-and-reach test required firefighters to reach as forward as far as possible on the ruler of a standardized sit-and-reach box. Cardiorespiratory fitness was estimated using the non-exercise method, using the following formula: oxygen consumption (VO_2max_) = 3.542 + (−0.014 × Age) + [0.015 × Body Mass (kg)] + (−0.011 × Resting Heart Rate) (Rexhepi and Brestovci, 2014). Relative VO_2max_ (relVO_2max_) was then calculated from the generated absolute VO_2max_ value. The estimated VO_2max_ formula was reported to be a moderately correlated predictor of VO_2max_ (r = 0.688, *p* < 0.001; $\bar{X}$ SD = 3.5420.314 vs. 3.5440.218). This was validated by Sun et al. (2022), who noted the formula was reliable in estimating VO_2max_ in men (R^2^ = 0.258; RMSE = 2.657; SEE = 0.051) and women (R^2^ = 0.213; RMSE = 2.202; SEE = 0.076), in the general population.

### 2.4 Musculoskeletal health measure summary

MSH encompassed MSIs and MSD. For MSIs, the Nordic Musculoskeletal Questionnaire (Crawford, 2007; Chairani, 2020) was used to acquire information on injuries and their location. The Cornell Musculoskeletal Discomfort Questionnaire (Hedge et al., 1999) was used to assess information on the location of discomfort.

### 2.5 Physical activity measures

Physical activity was assessed using the International Physical Activity Questionnaire (IPAQ) and using the questionnaire converted to weekly metabolic equivalent (MET) minutes (Bohlmann et al., 2001). Using the IPAQ cut-off values for physical activity levels, firefighters were further classified into highly active, which included firefighters who accumulated ≥3000 MET minutes a week of low-, moderate-, and vigorous-intensity MET minutes a week or ≥1,500 of vigorous-intensity MET minutes, only, a week (Bohlmann et al., 2001). Minimally active firefighters were classified as those who accumulated ≥600 MET minutes of low-, moderate-, and vigorous-intensity MET minutes a week. Insufficiently active firefighters were classified as those with <600 MET minutes a week (Bohlmann et al., 2001). In addition, physical activity was classified into total weekly MET minutes, total low-intensity physical activity minutes, total moderate-intensity physical activity minutes, and total vigorous-intensity physical activity minutes.

### 2.6 Statistical analysis

The data were analyzed using SPSS® software, version 28 (Chicago, Illinois, United States). The Shapiro–Wilk test was used to determine the distribution of the data, and the assumption of normal distribution was retained for the continuous variables of physical fitness and not normally distributed for measures of physical activity. Continuous variables of physical fitness are summarized as means and standard deviations, and continuous variables of physical activity are summarized as medians and 25th to 75th percentiles. Firefighters were classified into the following groups: those with 10-year age intervals, those with MSIs or those uninjured and injury location, and those with or without discomfort and location of discomfort. Group comparisons were based on independent t-tests and analysis of variance (ANOVA) for physical fitness parameters, and the Mann–Whitney *U* test for physical activity parameters. For the ANOVA analysis, Bonferroni correction was applied. Pearson’s correlation analysis was performed to determine the correlation between physical fitness and age, sex [point-biserial correlation (0 = males and 1 = females)], body mass index (BMI), and weekly MET minutes. Point-biserial correlations were also performed for dichotomous measures of MSH and continuous variables of age, BMI, and weekly MET minutes. In addition, chi-squared test was used to compare MSIs and MSD according to age groups. Univariable and multivariable logistic regressions were performed to determine the association between MSH parameters, which were treated as the outcome/dependent variable, and physical fitness, which designated the exploratory/independent variables. As exploratory variables, physical fitness was used as a continuous measure of physical fitness (abVO_2max_, relVO_2max_, grip and leg strength, push-ups, sit-ups, sit-and-reach, and lean body mass). Selection of exploratory variables used as covariates was evidence-based and based on a previous research study that consistently reported an association between MSIs and MSD in firefighters. Collinearity was assessed using the variance inflation factor (VIF) between the exploratory variables used in the adjusted models and deemed acceptable with a VIF of <5. In addition, to ensure autocorrelation was not present between independent variables, a correlation coefficient of <0.8 was used. Due to collinearity between age and years of experience, two separate multivariable models were used. Attributes adjusted for in model 2 included age, sex, BMI, and weekly MET minutes, and in model 3, years of experience was favored over age. A *p*-value of <0.05 was used to indicate statistical significance.

## 3 Results

Table 1 presents the physical fitness measures according to sex and age groups in firefighters. Weight (*p* < 0.001), BMI (*p* < 0.001), and weekly MET (*p* < 0.05) minutes were significantly different between age groups. All physical fitness measures were significantly different between the age groups, particularly abVO_2max_, relVO_2max_, sit-ups, push-ups, and sit-and-reach scores (*p* < 0.001). After Bonferroni correction, abVO_2max_, relVO_2max_, grip strength, leg strength sit-ups, push-ups, sit-and-reach, and LBM remained robust to adjustment. According to sex, male firefighters were taller, heavier, and stronger and had a higher abVO_2max_ and LBM. Female firefighters had a higher relVO_2max_ and were more flexible. Age was negatively correlated with abVO_2max_, relVO_2max_, leg strength, push-ups, sit-ups, sit-and-reach, and LBM (all *p* < 0.01). AbVO_2_max, grip strength, leg strength, and LBM were lower in female firefighters (all *p* < 0.01). BMI was negatively correlated with abVO_2max_, relVO_2max_, push-ups, sit-ups, and sit-and-reach and positively correlated with LBM (all *p* < 0.01). Weekly MET minutes were positively correlated to abVO_2max_, leg strength, and sit-and-reach (all *p* < 0.05). Most MSIs were reported in firefighters aged between 30 and 49 (*p* = 0.008), which was predominantly upper limb injuries (*p* = 0.010).

**TABLE 1:** Physical fitness parameters, musculoskeletal disorders, and musculoskeletal injuries according to age groups in firefighters.

	Age group								p					
	20–29		30–39		40–49		50+				Age	Sex	BMI	TWMETM
	N	$\bar{X}$ ±SD	N	$\bar{X}$ ±SD	N	$\bar{X}$ ±SD	N	$\bar{X}$ ±SD			r	rpb	r	r
Height (cm)	72	173.1 ± 7.4	95	173.1 ± 7.6	83	171.3 ± 8.2	58	174.7 ± 7.4	0.067		—	—	—	—
Weight (kg)	72	76.7 ± 12.5	95	80.6 ± 15.2	83	84.2 ± 14.3	58	91.1 ± 14.8	<0.001**		—	—	—	—
Body mass index (kgm-2)	72	25.5 ± 3.6	95	26.9 ± 4.9	83	28.7 ± 4.5	58	29.5 ± 4.7	<0.001**		—	—	—	—
Weekly MET minutes	72	3972.2 ± 2887.7	95	3224.7 ± 2955.3	83	2564.1 ± 2624.6	58	2759.9 ± 2756.7	0.014*		—	—	—	—
abVO_2_max (L•min)	72	3.5 ± 0.3	95	3.5 ± 0.3	83	3.3 ± 0.2	58	3.3 ± 0.3	<0.001**		−0.342**	0.138*	0.440**	0.172**
relVO_2_max (mL•kg•min)	72	46.8 ± 5.0	95	43.8 ± 5.7	83	40.4 ± 5.1	58	37.0 ± 4.1	<0.001**		−0.522**	−0.185**	−0.782**	0.102
Grip strength (kg)	72	89.6 ± 17.5	95	89.6 ± 17.5	82	84.8 ± 18.8	56	89.9±12.3	0.008**		−0.079	0.526**	0.016	0.024
Right grip strength (kg)	72	45.3 ± 9.0	95	47.3 ± 9.4	82	42.2 ± 9.9	56	45.7 ± 6.4	0.003**		−0.079	0.499**	0.043	0.051
Left grip strength (kg)	72	44.3 ± 8.9	95	46.6 ± 9.4	82	42.6 ± 9.4	56	44.2 ± 6.7	0.026*		−0.081	0.522**	0.030	0.039
Leg strength (kg)	72	124.1 ± 29.2	95	121.2 ± 27.8	82	106.4 ± 28.4	56	111.7 ± 28.8	<0.001**		−0.221**	0.406**	0.130*	0.225**
Push-ups (rpm)	72	38.9 ± 10.5	95	33.7 ± 11.9	82	27.5 ± 15.0	56	20.2 ± 9.9	<0.001**		−0.482**	0.099	−0.293**	0.138*
Sit-ups (rpm)	72	33.9 ± 6.9	95	30.9 ± 8.8	82	25.7 ± 11.3	56	20.5 ± 9.4	<0.001**		−0.450**	0.071	−0.375**	0.073
Sit-and-reach (cm)	72	45.6 ± 8.2	95	42.5 ± 9.3	82	43.6 ± 8.8	56	38.9 ± 9.4	<0.001**		−0.208**	−0.184**	−0.250**	0.116*
Lean body mass (kg)	72	58.6 ± 8.2	95	59.3 ± 9.5	83	60.4 ± 10.1	57	64.6 ± 9.9	0.002**		−0.163**	0.534**	0.333**	0.050

	Sex								p					
		Males		Females										
	N	$\bar{X}$ ± SD	N	$\bar{X}$ ± SD										
Height (cm)	275	174.3 ± 6.5	34	161.7 ± 7.4	—	—	—	—	<0.001**		—	—	—	—
Weight (kg)	275	83.3 ± 14.2	34	75.8 ± 18.8	—	—	—	—	0.005**		—	—	—	—
Body mass index (kgm-2)	275	27.4 ± 4.4	34	28.9 ± 6.4	—	—	—	—	0.064		—	—	—	—
Weekly MET minutes	275	3141.9 ± 2859.4	34	2991.2 ± 2800.9	—	—	—	—	0.772		—	—	—	—
abVO_2_max (L•min)	275	3.4 ± 0.3	34	3.3 ± 0.3	—	—	—	—	0.014*		—	—	—	—
relVO_2_max (mL•kg•min)	275	41.9 ± 5.9	34	45.1 ± 7.4	—	—	—	—	0.005**		—	—	—	—
Grip strength (kg)	270	92.9 ± 15.4	34	64.6 ± 12.0	—	—	—	—	<0.001**		—	—	—	—
Right grip strength (kg)	270	46.7 ± 8.2	34	32.7 ± 6.3	—	—	—	—	<0.001**		—	—	—	—
Left grip strength (kg)	270	46.1 ± 7.9	34	31.9 ± 6.1	—	—	—	—	<0.001**		—	—	—	—
Leg strength (kg)	270	120.6 ± 26.9	34	82.0 ± 23.9	—	—	—	—	<0.001**		—	—	—	—
Push-ups (rpm)	270	31.4 ± 14.3	34	26.7 ± 10.1	—	—	—	—	0.105		—	—	—	—
Sit-ups (rpm)	270	28.4 ± 10.4	34	25.9 ± 9.9	—	—	—	—	0.140		—	—	—	—
Sit-and-reach (cm)	270	42.3 ± 9.1	34	25.9 ± 9.9	—	—	—	—	0.006**		—	—	—	—
Lean body mass (kg)	275	62.4 ± 8.1	34	43.8 ± 5.7	—	—	—	—	<0.001**		—	—	—	—
Musculoskeletal health	N	%	N	%	N	%	N	%	p §		rpb	rpb	rpb	rpb
Musculoskeletal injury	19	14.6	39	31.0	41	31.5	31	23.8	0.008**		0.167*	0.119*	0.191*	-0.029
Upper limb	5	8.1	20	32.3	23	37.1	14	22.6	0.010*		0.149*	0.006	0.124*	0.033
Lower body	14	18.9	18	24.3	23	31.1	19	25.7	0.148		0.078	0.118*	0.137*	−0.019
Shoulder	1	5.9	4	23.5	6	35.3	6	35.3	0.124		0.132*	0.023	153*	−0.064
Lower back	1	4.2	10	41.7	8	33.3	5	20.8	0.139		0.080	0.015	0.082	−0.011
Ankle and foot	5	12.8	9	23.1	16	41.0	9	23.1	0.079		0.121*	-0.115*	0.154*	−0.026
Musculoskeletal discomfort	27	20.8	39	30.0	39	30.0	25	19.2	0.671		0.025	0.093	0.062	−0.015
Neck	6	18.2	11	33.3	11	33.3	5	15.2	0.727		0.014	0.002	−0.008	−0.026
Shoulder	6	14.3	16	38.1	12	28.6	8	19.0	0.471		0.042	0.105	−0.024	−0.028
Upper back	8	33.3	6	25.0	9	37.5	1	4.2	0.140		−0.071	−0.031	−0.083	0.010
Elbow	5	21.7	5	21.7	8	34.8	5	21.7	0.701		0.022	0.056	−0.034	0.029
Wrist and hand	5	16.1	10	32.3	13	41.9	3	9.7	0.157		−0.001	0.031	−0.004	0.098
Lower back	11	15.5	22	31.0	25	35.2	13	18.3	0.186		0.068	-0.014	0.135*	−0.007
Hip	4	21.1	6	31.6	6	31.6	3	15.8	0.959		0.005	−0.011	−0.061	−0.011
Thigh	4	22.2	5	27.8	7	38.9	2	11.1	0.636		−0.013	0.086	−0.027	−0.005
Ankle and foot	6	20.7	10	34.5	8	27.6	5	17.2	0.967		−0.002	−0.016	0.036	0.073

Note: * indicates statistical significance <0.05 and ** indicates statistical significance <0.01.

Correlations between physical fitness parameters and musculoskeletal health and age, sex, body mass index, and weekly MET minutes.

kg•m-2—kilogram per meter squared; L•min—liters per minute; mL•kg•min—milliliters per minute; kg—kilogram; rpm—repetitions per minute; $\bar{X}$ —mean; SD—standard deviation; p—significance level; %—percentage; r—Pearson’s correlation.; BMI—body mass index; TWMETM—total weekly metabolic equivalent minutes; ANOVA—indicates analysis of variance; §—indicates Chi-squared; rpb—point biserial correlation coefficient; rτ—Kendall’s tau coefficient.

Table 2 describes the MSH of firefighters according to demographic characteristics and physical activity classification in firefighters. Firefighters who were heavier (*p* = 0.006), older (*p* = 0.002), longer in service (*p* < 0.001), and with a higher BMI (*p* = 0.004) were more likely to report MSIs. Female firefighters were more likely to be injured (*p* = 0.038) than firefighters who were moderately active (*p* = 0.016).

**TABLE 2:** Musculoskeletal injuries and musculoskeletal discomfort according to demographic characteristics in firefighters.

	Injured		Never injured		p †	Musculoskeletal discomfort		Without musculoskeletal discomfort			p †
	N	$\tilde{X}$ (p25th–p75th)	N	$\tilde{X}$ (p25th–p75th)		N	$\tilde{X}$ (p25th–p75th)	N		$\tilde{X}$ (p25th–p75th)	
Height (cm)	130	172.9 (167.5, 178.0)	178	173.5 (169.0, 177.9)	0.512	130	173.0 (168.4, 178.0)	179		173.3 (168.0, 177.9)	0.942
Weight (kg)	130	82.4 (74.0, 95.9)	178	80.8 (72.1, 87.5)	0.006**	130	80.7 (71.9, 91.6)	179		82.0 (73.0, 90.1)	0.707
Age (years)	130	42.0 (32.0, 49.0)	180	36.0 (29.0, 46.0)	0.002**	130	39.0 (32.0, 46.5)	179		37.0 (29.0, 48.0)	0.478
Years of experience (years)	130	17.0 (7.0, 25.0)	178	11.0 (4.0, 19.0)	<0.001**	130	15.0 (6.8, 22.3)	179		13.0 (5.0, 22.0)	0.219
Body mass index (kg•m-2)	130	27.6 (25.1, 31.7)	178	26.8 (23.0, 29.6)	0.004**	130	27.0 (24.5, 31.1)	179		27.2 (23.9, 30.2)	0.469
Total weekly MET minutes	130	2000.5 (1176.8, 3600.0)	178	2348.5 (1073.3, 4819.5)	0.408	130	2009.5 (1167.0, 3743.0)	179		2400.0 (1160.0, 44.97.0)	0.474

	N	%	N	%	p §	N	%	N		%	p §
Sex											
Male	110	40.0	164	59.9	0.038*	112	40.7	163		59.3	0.174
Female	20	58.8	14	41.2		18	52.9	16		47.1	
Physical activity classification											
Vigorously active	15	29.4	36	70.6	0.016*	17	33.3	34		66.7	0.067
Moderately active	72	50.3	71	49.7		70	49.0	73		51	
Low active	43	37.7	71	62.3		43	37.4	72		62.6	

Note: * indicates statistical significance <0.05; ** indicates statistical significance <0.01.

cm—centimeter; kg—kilogram; kg•m-2—kilogram per meter squared; $\tilde{X}$ —median; p25th–p75th—25th percentile to 75th percentile; %—percent; †—Mann–Whitney U test, and §—indicates Chi-squared.

Table 3 presents the physical activity levels of firefighters according to age, years of experience, BMI, and weekly MET minutes and the physical activity level according to MSIs and MSD. Age (*p* = 0.049) and weekly MET minutes (*p* < 0.001) were significantly different between activity levels in firefighters. Firefighters who reported MSIs participated in less vigorous-intensity physical activity than those who never reported an injury (p = 0.004). Firefighters who reported more upper-body injures participated in less low-intensity physical activity (*p* = 0.044). Firefighters who experienced increased lower back discomfort participated in more low-intensity weekly physical activity (*p* = 0.002).

**TABLE 3:** Physical activity levels of firefighters according to age, years of experience, body mass index, weekly MET minutes, musculoskeletal discomfort, and musculoskeletal injury in firefighters.

		Insufficiently active (*N* = 51)		Minimally active (*N* = 143)		Highly active (*N* = 115)	*p*-value	
		$\tilde{X}$ (p25th–p75th)		$\tilde{X}$ (p25th–p75th)		$\tilde{X}$ (p25th–p75th)		
Age		39.0 (32.0, 47.0)		40 (32.0 48.0)		35.0 (28.0, 45.0)	0.049*	
Years of experience		15.0 (6.0, 22.0)		15.0 (6.0, 25.0)		10.0 (4.0, 19.0)	0.063	
Body mass index		27.0 (23.2, 30.9)		27.3 (24.2, 30.9)		26.8 (24.4, 29.8)	0.819	
Weekly MET minutes		99.0 (0.0, 419.0)		1812.0 (1257.0, 2280.0)		5088.0 (3798.0, 7560.0)	<0.001	

		TWMETM		TLIPAM		TMIPAM		TVIPAM
	N	$\tilde{X}$ (p25th–p75th)	N	$\tilde{X}$ (p25th–p75th)	N	$\tilde{X}$ (p25th–p75th)	N	$\tilde{X}$ (p25th–p75th)
Injured	130	2080.0 (1202.5, 3699.0)	68	120.0 (60.0, 345.0)	116	360.0 (240.0, 660.0)	21	240.0 (180.0, 720.0)
Uninjured	178	2580.0 (1578.0, 5310.0)	80	145.0 (62.5, 360.0)	139	480.0 (215.0, 660.0)	54	360.0 (180.0, 720.0)
*p*-value		0.589		0.372		0.281		0.004**
Upper body injury	62	2090.0 (1173.8, 3575.3)	39	120 (60.0, 390.0)	55	420.0 (240.0, 660.0)	10	450.0 (180.0, 975)
Uninjured	247	2268.0 (1160.0, 4464.0)	109	150.0 (60.0, 360.0)	201	420.0 (207.5, 660.0)	66	310.0 (180.0, 720.0)
*p*-value		0.976		0.044*		0.466		0.105
Lower body injury	74	1986.5 (1199.5, 3880.0)	32	135.0 (60.0, 262.50	68	360.0 (205.0, 645.0)	15	240.0 (180.0, 720)
Uninjured	235	2280.0 (1149.0, 4200.0)	116	123.0 (60.0, 360.0)	188	480.0 (240.0, 660.0)	61	360.0 (180.0, 720.0)
*p*-value		0.796		0.377		0.492		0.283
Shoulder injury	17	1584.0 (1074.0, 2549.0)	9	60.0 (30.0, 280.0)	15	330.0 (240.0, 490.0)	2	570.0 (180.0)
Uninjured	292	2279.0 (1174.0, 4293.0)	139	130.0 (60.0, 360.0)	241	420.0 (230.0, 660.0)	74	340.0 (180.0, 720.0)
*p*-value		0.134		0.902		0.725		0.235
Lower back injury	24	1816.0 (1002.0, 3775.1)	16	120.0 (60.0, 390.0)	22	295.0 (112.5, 573.8)	5	360.0 (130.0, 870.0)
Uninjured	285	2268.0 (1183.0, 4100.0)	132	135.0 (60.0, 360.0)	234	450.0 (240.0, 660.0)	71	320.0 (180.0, 720.0)
*p*-value		0.591		0.061		0.740		0.691
Ankle and foot injury	39	2080.0 (1080.0, 3474.0)	20	95.0 (60.0, 330.0)	36	375.0 (120.0, 625.0)	7	200.0 (90.0, 300.0)
Uninjured	270	2272.5 (1167.5, 4239.0)	128	128.0 (60.0, 360.0)	220	335.0 (240.0, 660.0)	69	360.0 (180.0, 720.0)
*p*-value		0.609		0.749		0.678		0.230
Overall discomfort	130	2080.0 (2607)	65	130.0 (60.0, 360.0)	117	380.0 (210.0, 630.0)	25	360 (180.0, 720.0)
No discomfort	179	2655.0 (1440.0, 4986)	83	120.0 (60.0, 360.0)	139	480.0 (240.0, 700.0)	51	320.0 (180.0,720.0)
*p*-value		0.474		0.512		0.463		0.060
Neck discomfort	33	1812.0 (1094.3, 3932.3)	16	85.0 (48.8, 555.0)	29	420.0 (240.0, 720.0)	7	240.0 (80.0, 720.0)
No discomfort	276	2277.5 (1181.3, 3993.3)	132	140.0 (60.0, 360.0)	227	420.0 (220.0, 660.0)	69	360.0 (180.0, 720.0)
*p*-value		0.474		0.894		0.639		0.601
Shoulder discomfort	17	1584.0 (1074.3, 2549.0)	9	60.0 (30.0, 280.0)	37	300.0 (160.0, 660.0)	10	450.0 (180.0, 720.0)
No discomfort	292	2279.0 (1174.5, 4293.0)	139	130 (60.0, 360)	219	450.0 (240.0, 660.0)	66	340.0 (180.0, 720.0)
*p*-value		0.511		0.954		0.839		0.968
Upper back discomfort	7	1320.0 (1040.0, 3306.0)	10	300.0 (27.5, 978.8)	22	365.0 (135.0, 675.0)	4	720.0 (517.5, 720.0)
No discomfort	302	2263.5 (2276.8, 4050.0)	138	123.0 (60.0, 360.0)	234	430.0 (240.0, 660.0)	72	285.0 (180.0, 720.0)
*p*-value		0.885		0.669		0.714		0.494
Elbow discomfort	23	2946.0 (1062.0, 4800.0)	12	150.0 (42.5, 633.8)	21	600.0 (242.4, 735.0)	4	720.0 (247.5, 720.0)
No discomfort	286	2216.0 (1176.8, 3979.8)	136	123.0 (60.0, 360.0)	235	420.0 (215.0, 640.0)	72	310.0 (180.0, 720.0)
*p*-value		0.626		0.627		0.149		0.482
Wrist and hand discomfort	31	2640.0 (1205.0, 6288.0)	15	120.0 (60.0, 780.0)	26	445.0 (240.0, 727.5)	7	420.0 (200.0, 720.0)
No discomfort	278	2177.0 (1159.5, 3896.3)	133	126 (60.0, 360.0)	230	420.0 (218.8, 640.0)	69	300.0 (180.0, 720.0)
*p*-value		0.172		0.912		0.522		0.574
Lower back discomfort	71	2019.0 (1149.0, 3840.0)	45	140.0 (60.0, 420.0)	62	372.0 (118.8, 720.0)	14	240.0 (90.0, 450.0)
No discomfort	238	2280.0 (1176.8, 4050.0)	103	120.0 (60.0, 360.0)	194	470.0 (240.0, 645.0)	62	360.0 (180.0, 720.0)
*p*-value		0.745		0.002**		0.855		0.203
Hip discomfort	4	2980.5 (598.5, 3891.8)	9	200.0 (45.0, 1012.5)	17	260.0 (117.5, 675.0)	4	720.0 (247.5, 720.0)
No discomfort	305	2238.0 (1165.0, 4086.5)	139	120.0 (60.0, 360.0)	239	440.0 (240.0, 660.0)	72	310.0 (180.0, 720.0)
*p*-value		0.765		0.845		0.749		0.812
Thigh discomfort	18	2273.0 (815.0, 5280.0)	8	400.0 (52.5, 1136.3)	15	292.5 (140.0, 720.0)	5	420.0 (120.0, 720.0)
No discomfort	291	2240.0 (1188.0, 3960.0)	140	120.0 (60.0, 360.0)	241	440.0 (240.0, 660.0)	71	320.0 (180.0, 720.0)
*p*-value		0.898		0.877		0.625		0.753
Ankle and foot discomfort	29	2640.0 (2160.0, 6264.0)	12	120.0 (60.0, 603.75)	25	360.0 (240.0, 1080.0)	7	720.0 (180.0, 720.0)
No discomfort	280	2216.0 (1162.5, 3877.3)	136	128.0 (60.0, 360.0)	231	420.0 (215.0, 640.0)	69	320.0 (180.0, 720.0)
*p*-value		0.283		0.512		0.416		0.690

Note: * indicates statistical significance <0.05; ** indicates statistical significance <0.01.

$\tilde{X}$ —median; p25th–p75th—25th percentile to 75th percentile; %—percentage; TWMETM—total weekly metabolic equivalent minutes; TLIPAM—total low-intensity physical activity minutes; TMIPAM—total moderate-intensity physical activity minutes; TVIPAM—total vigorous-intensity physical activity minutes; †—indicates Mann–Whitney U test.

Table 4 describes the MSI information according to physical fitness in firefighters. RelVO_2max_ (*p* = 0.002), push-up (*p* = 0.008) and sit-up (*p* = 0.005) capacity, and sit-and-reach (*p* = 0.015) were significantly different between firefighters who experienced an MSI. Grip strength was significantly different according to the location of injury (*p* = 0.044). RelVO_2max_ (*p* = 0.002), push-ups (*p* = 0.009), and sit-ups (*p* = 0.011) were significantly lower in firefighters who reported sustaining a shoulder injury. Grip strength (*p* = 0.021), sit-ups (*p* = 0.022), and sit-and-reach (*p* = 0.049) were significantly lower in firefighters who sustained ankle and foot injuries.

**TABLE 4:** Physical fitness parameters based on musculoskeletal injuries in firefighters.

	abVO_2_max (L•min)		relVO_2_max (L•min)		Grip strength (kg)		Leg strength (kg)		Push-ups (rpm)		Sit-ups (rpm)		Sit-and-reach (cm)		Lean body mass (kg)		
	N	$\bar{X}$ ± SD	N	$\bar{X}$ ± SD	N	$\bar{X}$ ± SD	N	$\bar{X}$ ± SD	N	$\bar{X}$ ± SD	N	$\bar{X}$ ± SD	N	$\bar{X}$ ± SD	N		$\bar{X}$ ± SD
Injured	129	3.4 ± 0.3	129	41.1 ± 5.8	126	87.8 ± 17.7	126	113.9 ± 17.7	126	28.9 ± 14.7	126	26.4 ± 10.6	126	41.5 ± 9.7	128		60.6 ± 10.5
Uninjured	179	3.4 ± 0.3	179	43.1 ± 6.2	177	91.1 ± 17.3	177	117.8 ± 27.2	177	32.1 ± 13.3	177	29.8 ± 9.9	177	43.8 ± 8.7	180		60.2 ± 9.4
*p*-value		0.142		0.002**		0.108		0.367		0.008**		0.005**		0.015*			0.525
Upper body injury	62	3.4±.03	62	40.4 ± 5.1	62	91.5 ± 17.3	61	116.2 ± 28.5	60	28.0 ± 15.2	60	25.8 ± 11.4	61	42.2 ± 10.0	62		61.8 ± 10.2
Uninjured	247	3.4±0.3	247	42.8 ± 6.3	243	89.3 ± 17.3	242	116.2 ± 28.5	243	31.6 ± 13.3	243	29.1 ± 10.0	243	43.1 ± 8.9	246		59.9 ± 9.5
*p*-value		0.904		0.007**		0.373		0.938		0.075		0.031*		0.433			0.180
Lower body injury	74	3.4 ± 0.3	74	41.5 ± 5.9	73	86.2 ± 17.4	73	113.9 ± 32.9	73	29.7 ± 13.2	73	27.0 ± 10.1	73	40.9 ± 9.9	74		59.7 ± 9.7
Uninjured	235	3.4 ± 0.3	235	42.6 ± 6.2	231	90.8 ± 17.4	230	117.1 ± 27.4	230	31.3 ± 13.9	230	28.9 ± 10.4	231	43.4 ± 8.8	234		60.6 ± 9.7
*p*-value		0.161		0.173		0.046*		0.387		0.190		0.190		0.039*			0.494
Shoulder injury	17	3.4 ± 0.3	17	37.9 ± 4.6	16	96.9 ± 25.6	16	120.9 ± 24.9	16	22.2 ± 10.9	16	22.0 ± 10.2	16	40.3 ± 12.6	17		64.6 ± 11.5
Uninjured	292	3.5 ± 0.3	292	42.6 ± 6.1	288	89.3 ± 16.9	287	116.1 ± 29.0	287	31.4 ± 13.8	287	28.8 ± 10.3	288	42.9 ± 8.9	291		60.1 ± 9.5
*p*-value		0.259		0.002**		0.088		0.514		0.009**		0.011*		0.417			0.060
Lower back injury	24	3.4 ± 0.3	24	41.1 ± 5.8	24	90.3 ± 13.4	24	114.5 ± 29.6	24	25.2 ± 15.4	24	25.8 ± 11.5	24	40.7 ± 11.1	24		61.9 ± 11.5
Uninjured	285	3.4 ± 0.3	285	42.4 ± 6.2	280	89.7 ± 17.8	279	116.5 ± 28.8	279	31.4 ± 13.5	279	28.6 ± 10.2	280	43.0 ± 8.9	284		60.2 ± 9.5
*p*-value		0.641		0.317		0.871		0.750		0.036*		0.197		0.226			0.407
Ankle and foot injury	39	3.4 ± 0.3	39	40.7 ± 6.2	39	83.7 ± 14.9	39	111.9 ± 32.2	39	27.1 ± 14.4	39	24.9 ± 10.8	39	40.2 ± 10.7	39		59.3 ± 10.6
Uninjured	270	3.4 ± 0.3	270	42.6 ± 6.1	265	90.6 ± 17.7	264	116.9 ± 28.3	264	31.4 ± 13.6	264	28.9 ± 10.2	265	43.2 ± 8.9	269		60.5 ± 9.6
*p*-value		0.527		0.071		0.021*		0.310		0.066		0.022*		0.049*			0.451

Note: * indicates statistical significance <0.05; ** indicates statistical significance <0.01; $\bar{X}$ —mean; SD—standard deviation; L•min—liters per minute; mL•kg•min—milliliters per minute; kg—kilogram; rpm—repetitions per minute.

Table 5 shows MSD and physical fitness parameters at various sites in firefighters. Higher levels of cardiorespiratory fitness, muscular endurance, and strength were related to firefighters reporting MSD. AbVO_2max_ was significantly different between those experiencing MSD and those without MSD (*p* = 0.038). A higher push-up capacity was related to neck discomfort (*p* = 0.019), shoulder discomfort (*p* = 0.047), upper back discomfort (*p* = 0.036), elbow and forearm discomfort (*p* = 0.015), wrist and hand discomfort (*p* < 0.001), hip discomfort (*p* = 0.043), thigh discomfort (*p* = 0.003), and ankle and foot discomfort (*p* = 0.039). Higher sit-up capacity was related to neck discomfort (*p* = 0.045), elbow and forearm discomfort (*p* = 0.022), wrist and hand discomfort (*p* = 0.027), and thigh discomfort (*p* = 0.006). Higher sit-and-reach score was related to lower neck discomfort (*p* = 0.029), elbow discomfort (*p* = 0.044), and thigh discomfort (*p* = 0.028).

**TABLE 5:** Physical fitness parameters based on the report of musculoskeletal discomfort at various sites.

Musculoskeletal discomfort	abVO_2_max (L•min)		relVO_2_max (L•min)		Grip strength (kg)		Leg strength (kg)		Push-ups (rpm)		Sit-ups (rpm)		Sit-and-reach (cm)		Lean body mass (kg)	
	N	$\bar{X}$ ± SD	N	$\bar{X}$ ± SD	N	$\bar{X}$ ± SD	N	$\bar{X}$ ± SD	N	$\bar{X}$ ± SD	N	± SD	N	$\bar{X}$ ± SD	N	$\bar{X}$ ± SD
Overall discomfort	130	3.4 ± 0.2	130	42.3 ± 6.1	127	88.4 ± 18.3	127	114.4 ± 29.5	127	31.7 ± 13.9	126	28.5 ± 11.0	127	42.4 ± 9.2	130	60.2 ± 9.7
No discomfort	179	3.3 ± 0.2	179	42.3 ± 6.2	177	90.7 ± 16.9	176	117.7 ± 28.4	177	30.3 ± 13.6	177	28.3 ± 9.9	177	43.1 ± 9.1	178	60.5 ± 9.7
*p*-value		0.038*		0.498		0.132		0.163		0.370		0.433		0.249		0.394
Neck discomfort	33	3.4 ± 0.3	33	42.2 ± 5.5	32	90.1 ± 14.0	32	116.9 ± 27.5	32	35.7 ± 12.8	32	29.2 ± 10.6	32	39.9 ± 8.2	33	60.7 ± 7.8
No discomfort	276	3.4 ± 0.3	276	42.3 ± 6.2	276	89.7 ± 17.9	276	116.2 ± 29.0	271	30.3 ± 13.8	271	28.3 ± 10.3	272	43.2 ± 9.2	275	60.3 ± 9.9
*p*-value		0.356		0.446		0.441		0.450		0.019*		0.334		0.029*		0.409
Shoulder discomfort	42	3.4 ± 0.3	42	42.7 ± 5.4	40	94.3 ± 18.0	40	120.3 ± 26.4	40	34.3 ± 12.8	40	31.0 ± 11.8	40	42.1 ± 9.4	42	62.6 ± 7.9
No discomfort	267	3.4 ± 0.3	267	42.4 ± 6.3	264	89.0 ± 17.3	263	115.7 ± 29.2	267	30.4 ± 13.9	263	28.0 ± 10.1	264	42.9 ± 9.1	266	60.0 ± 9.9
*p*-value		0.394		0.247		0.037*		0.176		0.047*		0.045*		0.286		0.054
Upper back discomfort	24	3.5 ± 0.3	24	44.5 ± 5.9	24	88.1 ± 15.1	24	109.9 ± 25.5	24	35.7 ± 12.2	24	32.5 ± 8.8	24	42.1 ± 9.4	24	59.1 ± 8.8
No discomfort	285	3.4 ± 0.3	285	42.1 ± 6.1	280	89.9 ± 17.7	279	115.9 ± 29.1	279	30.4 ± 13.8	279	28.1 ± 10.4	280	42.9 ± 9.1	284	60.5 ± 9.8
*p*-value		0.159		0.034*		0.314		0.127		0.036*		0.022*		0.333		0.253
Elbow discomfort	23	3.4 ± 0.3	23	42.2 ± 6.1	22	90.9 ± 14.1	22	117.1 ± 27.5	22	37.0 ± 11.9	22	31.8 ± 11.7	22	39.6 ± 7.7	23	61.5 ± 7.0
No discomfort	286	3.4 ± 0.3	286	42.3 ± 6.2	282	89.6 ± 17.7	281	116.3 ± 28.9	281	30.4 ± 13.8	281	28.2 ± 10.2	282	43.1 ± 9.2	285	60.3 ± 9.9
*p*-value		0.403		0.447		0.371		0.448		0.015*		0.055		0.044*		0.273
Wrist and hand discomfort	31	3.4 ± 0.3	31	41.9 ± 6.5	30	92.0 ± 13.1	30	120.9 ± 23.3	29	38.4 ± 14.5	30	31.9 ± 12.7	30	41.2 ± 9.8	31	60.4 ± 7.1
No discomfort	278	3.4 ± 0.3	278	42.4 ± 6.1	274	89.4 ± 17.9	273	115.8 ± 29.3	274	30.1 ± 13.5	273	28.0 ± 10.0	274	43.0 ± 9.1	277	60.3 ± 9.9
*p*-value		0.453		0.328		0.140		0.180		<0.001**		0.027*		0.146		0.478
Lower back discomfort	71	3.5 ± 0.3	71	41.6 ± 5.4	69	86.9 ± 14.8	69	111.6 ± 30.5	69	29.9 ± 14.4	68	27.4 ± 11.2	69	41.5 ± 9.3	71	60.3 ± 9.7
No discomfort	238	3.4 ± 0.3	238	42.5 ± 6.3	235	90.5 ± 18.2	235	117.7 ± 28.2	234	31.1 ± 13.6	235	28.7 ± 10.1	235	43.2 ± 9.1	237	60.4 ± 9.7
*p*-value		0.030*		0.134		0.067		0.061		0.262		0.187		0.084		0.462
Hip discomfort	19	3.4 ± 0.2	19	43.2 ± 6.3	19	90.8 ± 11.9	19	117.1 ± 25.4	19	36.1 ± 11.9	19	31.9 ± 9.4	19	40.8 ± 10.9	19	61.2 ± 9.5
No discomfort	290	3.4 ± 0.3	290	42.3 ± 6.1	285	89.6 ± 17.8	284	116.3 ± 29.1	284	30.5 ± 13.8	284	28.2 ± 10.4	285	42.99.0	289	60.3 ± 9.7
*p*-value		0.350		0.269		0.391		0.449		0.043*		0.062		0.163		0.347
Thigh discomfort	18	3.5 ± 0.2	18	42.2 ± 5.5	18	94.3 ± 10.6	18	125.1 ± 25.3	18	39.5 ± 12.4	18	34.3 ± 12.1	18	38.8 ± 9.3	18	63.4 ± 7.7
No discomfort	291	3.4 ± 0.3	291	42.3 ± 6.2	286	89.4 ± 17.8	286	115.8 ± 28.9	285	30.3 ± 13.7	285	28.0 ± 10.1	286	43.1 ± 9.1	290	60.2 ± 9.8
*p*-value		0.165		0.466		0.042*		0.092		0.003**		0.006**		0.028*		0.084
Ankle and foot discomfort	29	3.5 ± 0.3	29	41.7 ± 6.2	29	89.7 ± 14.1	29	118.1 ± 28.1	29	35.2 ± 13.3	28	30.3 ± 11.0	29	40.9 ± 9.9	29	60.3 ± 9.2
No discomfort	280	3.4 ± 0.3	280	42.4 ± 6.1	275	89.7 ± 17.8	274	116.1 ± 28.9	274	30.4 ± 13.7	275	28.2 ± 10.3	275	43.0 ± 9.1	279	60.4 ± 9.7
*p*-value		0.144		0.276		0.496		0.360		0.039*		0.158		0.115		0.478

Note: * indicates statistical significance <0.05; ** indicates statistical significance <0.01; $\bar{X}$ —mean; SD—standard deviation; L•min—liters per minute; mL•kg•min—milliliters per minute; kg—kilogram; rpm—repetitions per minute.

Table 6 presents the association between physical fitness and MSIs in firefighters. Univariate analysis indicated that a higher abVO_2max_ (*p* = 0.005), push-up (*p* = 0.017) and sit-up capacity (*p* = 0.006), and sit-and-reach (*p* = 0.034) were negatively associated with firefighters reporting MSIs. RelVO_2max_ (*p* = 0.007) and sit-up capacity (*p* = 0.032) were negatively associated with firefighters reporting upper body MSIs. An increase in grip strength (*p* = 0.048) and sit-and-reach (*p* = 0.041) was negatively associated with firefighters reporting lower body MSIs. None of the variables was associated with MSIs after adjustment for covariates. An increase in relVO_2max_ (*p* = 0.003), push-ups (*p* = 0.011), and sit-ups (*p* = 0.013) was negatively associated with firefighters reporting a shoulder injury. Push-ups were significantly and negatively associated with lower back injuries (*p* = 0.039). An increase in grip strength (*p* = 0.022), sit-ups (*p* = 0.024), and sit-and-reach (*p* = 0.048) was negatively associated with firefighters reporting ankle and foot injuries. After adjustment for covariates, none of the exploratory variables remained significant.

**TABLE 6:** Association between physical fitness and musculoskeletal injuries in firefighters.

	Univariate models		Multivariate models			
	Model 1		Model 2^a^		Model 3^b^	
	OR (95% CI)	*p*-value	OR (95% CI)	*p*-value	OR (95% CI)	*p*-value
Dependent variable: musculoskeletal injuries						
abVO_2max_ (L•min)	1.85 (0.81, 4.19)	0.143	—	—	—	—
relVO_2max_ (mL•kg•min)	0.95 (0.91, 0.98)	0.005**	1.01 (0.93, 1.09)	0.909	1.02 (0.94, 1.11)	0.644
Grip strength (kg)	0.99 (0.98, 1.00)	0.109	—	—	—	—
Leg strength (kg)	0.99 (0.99, 1.00)	0.356	—	—	—	—
Push-ups (rpm)	0.98 (0.96, 0.99)	0.017*	0.99 (0.97, 1.01)	0.994	1.00 (0.98, 1.02)	0.869
Sit-ups (rpm)	0.97 (0.95, 0.99)	0.006**	0.99 (0.96, 1.01)	0.365	0.99 (0.96, 1.02)	0.505
Sit-and-reach (cm)	0.97 (0.95, 0.99)	0.034*	0.98 (0.95, 1.01)	0.153	0.98 (0.95, 1.01)	0.182
Lean body mass (kg)	1.01 (0.98, 1.03)	0.524	—	—	—	—
Dependent variable: upper body injuries						
abVO_2max_ (L•min)	1.06 (0.393, 2.88)	0.903	—	—	—	—
relVO_2max_ (mL•kg•min)	0.94 (0.89, 0.98)	0.007**	0.96 (0.87, 1.06)	0.407	0.95 (0.86, 1.05)	0.289
Grip strength (kg)	1.01 (0.99, 1.02)	0.372	—	—	—	—
Leg strength (kg)	1.00 (0.99, 1.01)	0.938	—	—	—	—
Push-ups (rpm)	0.98 (0.96, 1.00)	0.076	—	—	—	—
Sit-ups (rpm)	0.97 (0.94, 0.99)	0.032*	0.99 (0.96, 1.02)	0.442	0.99 (0.95, 1.02)	0.342
Sit-and-reach (cm)	0.99 (0.96, 1.02)	0.432	—	—	—	—
Lean body mass (kg)	1.02 (0.99, 1.05)	0.181	—	—	—	—
Dependent variable: lower body injuries			—	—	—	—
abVO_2max_ (L•min)	1.97 (0.763, 5.09)	0.161	—	—	—	—
relVO_2_max (mL•kg•min)	0.97 (0.93,1.01)	0.173	—	—	—	—
Grip strength (kg)	0.99 (0.97, 1.00)	0.048*	1.01 (0.99, 1.03)	0.203	0.99 (0.97, 1.01)	0.405
Leg strength (kg)	0.99 (0.99, 1.01)	0.415	—	—	—	—
Push-ups (rpm)	0.99 (0.97, 1.01)	0.386	—	—	—	—
Sit-ups (rpm)	0.98 (0.96, 1.01)	0.190	—	—	—	—
Sit-and-reach (cm)	0.97 (0.94, 0.99)	0.041*	0.97 (0.94, 1.00)	0.092	0.98 (0.95, 1.01)	0.124
Lean body mass (kg)	0.99 (0.96, 1.02)	0.493	—	—	—	—
Dependent variable: shoulder injuries						
abVO_2max_ (L•min)	2.82 (0.48, 16.72)	0.253				
relVO_2max_ (mL•kg•min)	0.87 (0.79, 0.95)	0.003**	0.86 (0.71, 1.03) §	0.105	0.83 (0.69, 1.01)	0.060
Grip strength (kg)	1.03 (0.99, 1.06)	0.090	—	—	—	—
Leg strength (kg)	1.01 (0.99, 1.02)	0.521	—	—	—	—
Push-ups (rpm)	0.95 (0.91, 0.99)	0.011*	0.97 (0.92, 1.01)	0.158	0.96 (0.92, 1.01)	0.105
Sit-ups (rpm)	0.94 (0.89, 0.99)	0.013*	0.97 (0.91, 1.03)	0.264	0.96 (0.91, 1.02)	0.167
Sit-and-reach (cm)	0.97 (0.92, 1.02)	0.251	—	—	—	—
Lean body mass (kg)	1.05 (0.99, 1.11)	0.063	—	—	—	—
Dependent variable: lower back injuries						
abVO_2max_ (L•min)	1.45 (0.32, 6.47)	0.629				
relVO_2max_ (mL•kg•min)	0.97 (0.90, 1.04)	0.321				
Grip strength (kg)	1.00 (0.98, 1.03)	0.878				
Leg strength (kg)	0.99 (0.98, 1.01)	0.738				
Push-ups (rpm)	0.97 (0.94, 0.99)	0.039*	0.97 (0.94, 1.01)	0.157	0.98 (0.95, 1.02)	0.277
Sit-ups (rpm)	0.97 (0.94, 1.01)	0.199				
Sit-and-reach (cm)	0.97 (0.93, 1.02)	0.219				
Lean body mass (kg)	1.02 (0.98, 1.06)	0.405				
Dependent variable: ankle and foot injuries						
abVO_2max_ (L•min)	1.49 (0.45, 5.02)	0.513				
relVO_2max_ (mL•kg•min)	0.95 (0.89, 1.01)	0.074				
Grip strength (kg)	0.98 (0.96, 0.99)	0.022*	0.98 (0.96, 1.01)	0.135	0.99 (0.96, 1.01)	0.235
Leg strength (kg)	0.99 (0.98, 1.01)	0.300				
Push-ups (rpm)	0.98 (0.95, 1.00)	0.070				
Sit-ups (rpm)	0.96 (0.933, 0.99)	0.024*	0.99 (0.95, 1.03)	0.522	0.99 (0.96, 1.04)	0.942
Sit-and-reach (cm)	0.96 (0.93, 1.00)	0.048*	0.97 (0.93, 1.01)	0.134	0.97 (0.94, 1.01)	0.194
Lean body mass (kg)	0.99 (0.95, 1.02)	0.453				

Note: * indicates statistically significance <0.05; ** indicates statistical significance <0.01.

a —covariates adjusted for: age, sex, body mass index, and weekly MET minutes; b—covariates adjusted for: years of experience, sex, body mass index, and weekly MET minutes; L•min—liters per minute; mL•kg•min—milliliters per minute; kg—kilogram; rpm—repetitions per minute; §—significant when adjusted for age and sex only; §—significant when adjusted for years of experience and sex only.

Table 7 presents the association between physical fitness and MSD in firefighters. A higher push-up capacity was positively associated with neck discomfort (*p* = 0.038), elbow and forearm discomfort (*p* = 0.031), wrist and hand discomfort (*p* = 0.002), and thigh discomfort (*p* = 0.007). A higher sit-up capacity was positively associated with upper back discomfort (*p* = 0.045) and thigh discomfort (*p* = 0.013). In model 2, after adjustments for age, sex, BMI, and weekly MET minutes, a one-unit increase in push-up capacity increased the odds of neck discomfort, elbow discomfort, wrist and hand discomfort, and thigh discomfort by 5%, 5%, 6%, and 6%, respectively, and that in sit-up capacity increased the odds of MSD by 9%. In model 3, after adjustments for years of experience, sex, BMI, and weekly MET minutes, a one-unit increase in push-up capacity increased the odds of neck discomfort, elbow discomfort, ankle discomfort, and foot discomfort by 4%, 5%, 6%, and 7%, respectively. A one-unit increase in sit-up capacity increased the odds of reporting thigh discomfort by 8%.

**TABLE 7:** Association between physical fitness and musculoskeletal discomfort in firefighters.

	Univariate models			Multivariate models			
	Model 1			Model 2^a^		Model 3^b^	
	OR (95% CI)	*p*-value		OR (95% CI)	*p*-value	OR (95% CI)	*p*-value
Exploratory variable: abVO_2max_ (L•min)							
Musculoskeletal discomfort	2.10 (0.92, 4.79)	0.077		—	—	—	—
Lower back discomfort	2.52 (0.96, 6.67)	0.062		—	—	—	—
Exploratory variable: grip strength (kg)							
Shoulder discomfort	1.02 (0.99, 1.04)	0.075		—	—	—	—
Thigh discomfort	1.02 (0.99, 1.04)	0.249		—	—	—	—
Exploratory variable: push-ups (rpm)							
Neck discomfort	1.03 (1.00, 1.06)	0.039*		1.04 (1.01, 1.07)	0.014*	1.04 (1.01, 1.08)	0.011*
Shoulder discomfort	1.02 (0.99, 1.05)	0.094		—	—	—	—
Upper back discomfort	1.03 (0.99, 1.06)	0.074		—	—	—	—
Elbow discomfort	1.04 (1.00, 1.07)	0.031*		1.05 (1.01, 1.08)	0.019**	1.05 (0.99, 1.09)	0.016*
Wrist and hand discomfort	1.05 (1.02, 1.08)	0.002**		1.06 (1.02, 1.09)	0.001**	1.06 (1.00, 1.09)	<0.001
Hip discomfort	1.03 (0.99, 1.07)	0.088		—	—	—	—
Thigh discomfort	1.05 (1.01, 1.09)	0.007**		1.06 (1.02, 1.11)	0.005**	1.07 (1.03, 1.12)	0.002**
Ankle and foot discomfort	1.03 (0.99, 1.06)	0.790		—	—	—	—
Exploratory variable: sit-ups (rpm)							
Shoulder discomfort	1.03 (0.99, 1.06)	0.091		—	—	—	—
Upper back discomfort	1.05 (1.00, 1.09)	0.045*		1.04 (0.99, 1.09)	0.143	1.04 (0.99, 1.09)	0.135
Wrist and hand discomfort	1.04 (0.99, 1.08)	0.055		—	—		
Thigh discomfort	1.07 (1.01, 1.12)	0.013*		1.09 (1.03, 1.51)	0.003**	1.08 (1.02, 1.14)	0.005**
Exploratory variable: sit-and-reach (cm)							
Neck discomfort	0.96 (0.924, 1.00)	0.060		—	—	—	—
Elbow discomfort	0.96 (0.92, 1.01)	0.090		—	—	—	—
Thigh discomfort	0.95 (0.903, 1.00)	0.058		—	—	—	—

Note: * indicates statistical significance <0.05; ** indicates statistical significance <0.01.

a—covariates adjusted for age, sex, body mass index, and weekly MET minutes; b—covariates adjusted for years of experience, sex, body mass index, and weekly MET minutes; L•min—liters per minute; mL•kg•min—milliliters per minute; kg—kilogram; rpm—repetitions per minute.

## 4 Discussion

In this study, we found that firefighters with a higher level of physical fitness reported fewer musculoskeletal injuries. However, higher levels of physical fitness were also associated with increased odds of MSD. Several studies have found that a higher level of physical fitness may reduce the likelihood of MSIs, which is similar to the results of the present study, likely due to the increase in bone mineral density, connective tissue health, muscle mass, and improved balance and coordination (Hong et al., 2012; Poplin et al., 2013; Poplin et al., 2016). The improvements in bone and soft tissue health, as a result of physical activity and increased physical fitness, may increase the volume of physical workload needed to cause a progressive decrease in MSH, which would lead to sudden MSIs on duty. In the current study, fitter firefighters may have reported higher levels of MSD due to overload in workload and insufficient/inadequate recovery or rest following the workload. This hypothesis is supported by the results showing physical activity levels were higher in firefighters who reported MSD, particularly those who were vigorously active. It is also possible that some firefighters experienced MSD due to a high level of physical activity in their leisure time, especially when off-duty, and high levels of occupational activity when on-duty. This persistent overload may predispose firefighters to pain and inflammation, possibly leading to MSD, and possibly, MSIs in firefighters (Vaulerin et al., 2016; Ras and Leach, 2022).

We found that an increase in relVO_2_max, push-ups, sit-ups, and sit-and-reach decreased the odds of firefighters reporting MSIs. Nowak et al. (2018) reported that firefighters who previously experienced an MSI had a lower push-up and sit-up capacity and lower cardiorespiratory capacity than those without an MSI. In addition, measures of explosive power were also higher in firefighters without injuries than in those who had previous injuries (Nowak et al., 2018). Similarly, Poplin et al. (2013) reported that higher cardiorespiratory capacity was associated with lower incidence of injuries in firefighters. Another study by Poplin et al. (2016) reported that lower levels of physical fitness increased the odds of firefighters sustaining an injury over a 5-year period. Two systematic reviews conducted by de la Motte et al. (2017); Lisman et al. (2017) support the results of the current study, reporting that higher cardiorespiratory fitness and muscular endurance were associated with lower incidences of MSIs. Injury-related absenteeism and the medical expenses associated with it are costly to fire departments, with most of these injuries being related to sprains and strains (Poston et al., 2011; Frost et al., 2016). Physical activity is essential in the strengthening and thickening of connective tissues, an increase in bone mineral density, and improvements in muscle endurance and strength (de la Motte et al., 2017; Lisman et al., 2017), which is likely in firefighters with higher physical fitness levels, reducing the overall reported MSIs seen in the current study. Moreover, studies have suggested that a substantial source of MSIs in firefighters is related to overexertion while engaged in fire suppression and other emergency situations (Frost et al., 2015a; Nowak et al., 2018; Le et al., 2020). Higher levels of physical fitness, particularly muscular strength and endurance, may increase the level of physical exertion needed to induce muscular and cardiorespiratory fatigue that leads to overexertion, thereby providing a protective effect on the musculoskeletal and cardiovascular systems (Henderson et al., 2007; Yu et al., 2015; Nowak et al., 2018; Le et al., 2020). Firefighters who have a higher level of physical fitness perform their duties with more efficiency and rigor. Thus, physical fitness and physicality are integral for firefighters’ occupational performance (Williford et al., 1999; Rhea et al., 2004; Chizewski et al., 2021) and injury prevention (Poplin et al., 2012; Poplin et al., 2013; Vaulerin et al., 2016; Nowak et al., 2018). To ensure the highest occupational efficiency, firefighters should maintain all measures of physical fitness through regular physical activity (Durand et al., 2011; Yu et al., 2015; Nowak et al., 2018). In addition, it is well-documented that cardiorespiratory fitness, muscular strength, and endurance decline as firefighters age, due, in part, to a lack of leisure time physical activity and the natural decline in MSH as a product of the aging process (Baur et al., 2012; Punakallio et al., 2012; Walker et al., 2014; Perroni et al., 2015; Frontera, 2017). This predisposes the firefighters to MSIs, especially, if they lack the necessary levels of physical fitness needed for firefighting. Adequate levels of cardiorespiratory fitness, muscle strength, endurance, and muscular function are important for injury prevention and job performance in firefighters (Smith, 2011; Poplin et al., 2013; Nowak et al., 2018). Furthermore, physical activity has been shown to promote the release of myokines from muscle tissue (Hamrick, 2011; Lee and Jun, 2019). Myokines play an important role in stress response and coordinating both positive and negative musculoskeletal changes to exercise and/or work (Hamrick, 2011; Lee and Jun, 2019). This may, further, support that more physically active and, subsequently, more physically fit firefighters are less likely to sustain MSIs.

The present study showed that higher relVO_2max_ and sit-up capacity were associated with lower odds of firefighters reporting upper limb injuries. In addition, an increase in relVO_2max_, push-up and sit-up capacity was associated with lower odds of firefighters reporting shoulder injuries and an increase in push-up capacity reduced the odds of firefighting reporting lower back injuries. Previous studies noted that people involved in occupations that require repetitive upper body motions are particularly susceptible to an increase in upper limb injuries (Ranney et al., 1995; Latko et al., 1999). An increase in physical fitness, particularly upper body muscular endurance capacity, may increase the workload needed to lead to overexertion-related shoulder injuries (de la Motte et al., 2017), especially as many firefighting-related duties encompass repetitive upper body movements (Frost et al., 2015a; Nowak et al., 2018). Cady et al. (1979) reported that higher physical fitness, which encompassed a prediction model that included cardiorespiratory endurance, flexibility, muscular strength, and diastolic blood pressure, was associated with lower back injuries in firefighters. Previous studies noted that exercise increases muscular strength and endurance, which could, potentially, reduce the likelihood of lower back injuries in firefighters (Taylor et al., 2014; Mayer et al., 2015; Moon et al., 2015). Similarly, Peate et al. (2007) reported that after an exercise intervention, improvements in abdominal strength and flexibility reduced the incidence of injuries in firefighters. Although not directly related, higher levels of muscular endurance may positively assist firefighters in reducing the incidence of injuries to their upper limbs and trunk, likely due to these areas having an increased stability and higher capacity to tolerate forceful repetitive movements (Cady et al., 1979; Beaton et al., 2002; Peate et al., 2007; de la Motte et al., 2017). The results of the current study indicated that for every 1 kg increase in grip strength and 1 cm increase in sit-and-reach score, there were lower odds of firefighters reporting lower limb injuries by 1% and 3%, respectively. A study reported that a higher sit-and-reach test score was associated with lower incidences of of MSIs (Lisman et al., 2017) and is likely related to a greater range of motion and a lower likelihood of stretching connective tissues to an uncomfortable degree. Similarly, Frost et al. (2015b) reported that push-up, deep squat, and sit-and-reach tasks in the functional movement screening significantly predicted injury status in firefighters. This was supported by Butler et al. (2013) who reported that the sit-and-reach score was a significant predictor of injury status in firefighters.

In the present study, with the introduction of age, sex, years of experience, BMI, and physical activity levels in the multivariate models, the significant associations were removed from all significant outcomes for MSIs. This suggests that although higher levels of physical fitness are necessary to protect firefighters from sustaining injuries, there are additional components that form part of a larger system of factors that also contribute to MSI prevention, which is supported by previous research (Nabeel et al., 2007; Vaulerin et al., 2016; Nowak et al., 2018; Ras and Leach, 2022). Firefighters remaining physically active to meet a minimum level of health-related physical fitness may be a prerequisite in reducing MSIs, but beyond this level may lead to chronic pain and injury (Poplin et al., 2016; Vaulerin et al., 2016; Lentz et al., 2019; Ras and Leach, 2022). This was seen in the present data, where firefighters who were more vigorously active reported less MSIs. However, similarly, firefighters who experienced MSD tended toward being more physically active, particularly vigorously active, as well. Although there were instances where this tended toward significance, statistical significance was not seen, perhaps due to the relatively small numbers of firefighters experiencing MSD. Previous studies have reported that older firefighters, with more years of experience, and who were heavier and more physically inactive, were particularly susceptible to sustaining MSIs while on duty (Poston et al., 2011; Jahnke et al., 2013; Phelps et al., 2018; Nazari et al., 2020b; Hollerbach et al., 2020). Moreover, studies found women were especially susceptible to MSIs, due to multiple factors, such as poor fitting equipment, lower muscle mass, particularly in the upper limbs, and lower bone mineral density (Sinden et al., 2013; McQuerry et al., 2019; Song et al., 2019). After adjusting for age, sex, and BMI in the multivariate analysis, we noted that our results no longer achieved significance. This was also noted in correlations where age, sex, and BMI were positively correlated to MSIs in firefighters. In addition, it is likely that due to working as a firefighter for longer periods, regardless of their fitness levels, these firefighters were more likely to sustain an injury during their career (Poston et al., 2011; Hong et al., 2012; Frost et al., 2015a).

We found that push-up capacity was significantly associated with increased odds of firefighters reporting discomfort in the neck, elbow and forearm, wrist and hand, and thigh regions. In addition, an increase in sit-up capacity was associated with an increase in the odds of firefighters reporting upper back and thigh discomfort. Rintala et al. (2015) reported that fitter pilots flew their aircrafts at speeds that induce higher acceleration speeds and physical workloads and, due to this higher workload, reported more symptoms of musculoskeletal pain, but fewer musculoskeletal disabilities. This may also relate to the firefighting profession, where fitter firefighters may perform their duties with greater rigor, power, and force (Williford et al., 1999; Chizewski et al., 2021), which may overload the firefighter’s musculoskeletal system. These fitter firefighters might be engaged in more vigorous-intensity work at fire or emergency scenarios, compared to their less fit counterparts, which leads to MSD. In addition, if firefighters participate in regular vigorous-intensity leisure-time physical activity, this may exacerbate an already strained musculoskeletal system, or overload the musculoskeletal system, leading to burnout, that increases the risk of MSIs (Vaulerin et al., 2016; Ras and Leach, 2022). This might cause additional MSD in firefighters, but have a positive effect on reducing MSIs, as seen in the current results. However, managing the overall workload may be key to maintaining MSH and reducing injury incidence in firefighters. Previous research has noted that monitoring overall workload is important for firefighters (Poplin et al., 2013; Vaulerin et al., 2016; de la Motte et al., 2017; Ras and Leach, 2022). This could provide a possible explanation to why fitter firefighters were more likely to report MSD, especially if the MSD caused by high workloads could, eventually, lead to overuse injury (Vaulerin et al., 2016). Lusa et al. (2015) reported that firefighters who reported sleep disturbances had chronic low back pain symptoms (Halson, 2008). Abbasi et al. (2018) reported that firefighters who were heavily physically active had poorer sleep quality and were more likely to report MSDs. Due to sleep being integral to recovery, this may provide an explanation as to why fitter firefighters experienced more MSD in the current results. In contrast to the current results, Nabeel et al. (2007) reported that higher levels of physical fitness were associated with a significant decrease in the incidence of chronic pain in police officers. Similarly, Beaton et al. (2002) reported that neck, back, and shoulder pain was significantly lower in firefighters who participated in more frequent aerobic exercise. It may be that MSD is an indication of excessive workload or insufficient recovery, which has been supported in other populations, such as nurses, paramedics, surgeons, and welders (Menzel et al., 2004; Tam and Yeung, 2006; Szeto et al., 2009; Shahriyari et al., 2020). However, this area is understudied in firefighters, and the findings are not particularly intuitive. Investigating MSD may provide valuable insight into MSH of firefighters and how this may eventually lead, or predispose, firefighters to injury. It is recommended that more research be conducted in this area to better understand the causal mechanisms between physical fitness and MSD, and the implications of MSD for the likelihood of sustaining an injury.

### 4.1 Strengths and limitations

This was the first study examining the relationship between physical fitness and MSH in CoCTFRS firefighters, a demographic that has received little attention with respect to scientific research. The study used validated instruments and trained researchers who objectively assessed the markers of physical fitness, except cardiorespiratory fitness (Ras et al., 2022c). Validated questionnaires were used to assess MSH. This work contributes unique information to a field of study that has not yet received adequate attention, particularly in a South African setting. The present study, however, has some limitations. The study’s cross-sectional design prevents the inference of causal associations. The study estimated relative and absolute cardiorespiratory fitness using a non-exercise calculation. The under-representation of female firefighters limits the generalizability of results to female firefighters. Although the study had a relatively large sample size, the low number of firefighters with MSD and MSIs limits the power of the statistical analysis.

## 5 Conclusion

The findings of the present study emphasize the need for firefighters maintaining high levels of physical fitness to lessen the risk of MSIs, particularly cardiorespiratory fitness, muscular endurance, and flexibility. In addition, our finding of a positive association between physical fitness and MSD indicates that care must be taken to implement well-structured fitness programs that take into account the need for adequate rest and recovery. This research highlights the importance of maintaining and/or improving physical fitness on MSH in firefighters, in the CoCTFRS, thus emphasizing the need for policy change. It is recommended that occupational health and safety professionals, as well as policymakers, ensure that firefighters participate in regular physical activity that is monitored for total weekly workload to reduce the likelihood of overexertion and ensure adequate recovery, and maintain an ideal level of health-related physical fitness to aid their occupational wellbeing. Furthermore, the development of workload guidelines is needed to further support the physical fitness requirements of firefighting and reduce the likelihood of MSIs and discomfort in firefighters. In future research, longitudinal studies are warranted to evaluate a potential causal relationship between physical fitness and improvements or decrements on the incidence of MSIs and, especially, MSD as this area is understudied with respect to firefighters. Although female firefighters represent a relatively small proportion of firefighters in the CoCTFRS, a larger and more representative sample of female firefighters should be included in future studies to allow for the generalizability of results to the female firefighter population.

## Data Availability

The original contributions presented in the study are included in the article/Supplementary Material. Further inquiries can be directed to the corresponding author.
